# Advanced Research on the Antioxidant Activity and Mechanism of Polyphenols from *Hippophae* Species—A Review

**DOI:** 10.3390/molecules25040917

**Published:** 2020-02-19

**Authors:** Mingyue Ji, Xue Gong, Xue Li, Congcong Wang, Minhui Li

**Affiliations:** 1 Department of Pharmacy, Baotou Medical College, Baotou 014060, China; Jimingyue9@163.com (M.J.); gongxue_2017@yeah.net (X.G.); Wangcongcong0@163.com (C.W.); 2 Department of Pharmacy, Inner Mongolia Medical University, Hohhot 010110, China; lixue950119@163.com; 3 Qiqihar Medical University, Qiqihar 161006, China; 4 Pharmaceutical Laboratory, Inner Mongolia Autonomous Region Academy of Chinese Medicine, Hohhot 010020, China; 5 Inner Mongolia Key Laboratory of Characteristic Geoherbs Resources Protection and Utilization, Baotou Medical College, Baotou 014060, China

**Keywords:** *Hippophae* species, polyphenols, antioxidant activity, applications

## Abstract

Oxidation is a normal consequence of metabolism in biological organisms. The result is the formation of detrimental reactive oxygen species (ROS) and reactive nitrogen species (RNS). A large number of studies have shown that polyphenolic compounds have good antioxidant properties. *Hippophae* species plants have high polyphenolic content and are widely used in food, medicinal, or the cosmetic field. The main polyphenols in *Hippophae* species are flavonoids, phenolic acids and tannins, which have multiple effects. However, there is a limited number of studies on polyphenols in *Hippophae* species plants. This review systematically summarizes the polyphenols compounds and antioxidant activity of *Hippophae* species plants, and it is noteworthy that the main mechanisms of the polyphenols of *Hippophae* with antioxidant activity have been summarized as follows: regulating enzyme activity, affect the antioxidant reaction of cells, and others. This review provides useful information for the further study and application of *Hippophae* species polyphenols and their antioxidant activity.

## 1. Introduction

Oxidation is a natural consequence of metabolism in biological organisms. The result is the formation of detrimental reactive oxygen species (ROS) and reactive nitrogen species (RNS), such as superoxide, hydrogen peroxide, singlet oxygen and nitric oxide radicals. Normally, the antioxidant system in the human body can scavenge these radicals, thereby maintaining the balance between oxidation and antioxidation. However, when the body cannot eliminate excessive ROS using the intracellular antioxidant enzyme system and extracellular antioxidant compounds, oxidative stress will occur, leading to chronic and degenerative diseases, such as osteoarthritis, atherosclerosis, cancer and other degenerative diseases related to aging [1,2,3].

**Figure 1:**
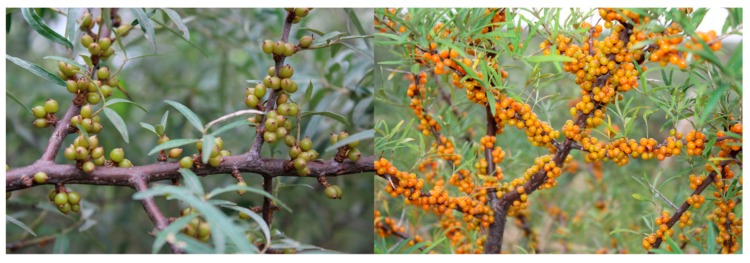
The fruit of sea buckthorn (*H. rhamnoides* L.).

*Hippophae* is a member of the Elaeagnaceae family, in which 7 species and 11 subspecies have been identified worldwide [4]. *Hippophae* is widely used in various fields, such as the food, medicine and cosmetic industries. *Hippophae rhamnoides* L., the representative plant of the genus *Hippophae*, can be used as a food and medicine [5] (Figure 1). Studies have shown that various nutrients and bioactive components are present in *Hippophae*. These include minerals, vitamins, polysaccharides, unsaturated fatty acid, terpenoids, polyphenolic compounds, nonsteroidal compounds, flavonoids, organic acids and volatile components, which have antioxidant, anti-inflammatory, anti-radiation and curative effects on burns and ulcers [6,7]. Polyphenols are secondary plant metabolites, arising from phenylalanine or shikimic acid, playing a pivotal role in counteracting various type of stress, other than contributing to the organoleptic properties of plants and plant-derived food [8]. In addition, it is well known that polyphenols exert beneficial effects upon human health, due to their antioxidant, anti-inflammatory, cardioprotective, anticancer and antimicrobial properties [9,10]. The antioxidant activity of sea buckthorn (*H. rhamnoides* L.) is mainly due to the redox characteristics of polyphenolic compounds, which mainly contain flavonoids, phenolic acids and tannins. In particular, kaempferol, quercetin, isorhamnetin, catechin, procyanidins and gallic acid are commonly present as bioactive compounds [11]. *H*. *rhamnoides* L. is a good source of active phenols for human consumption, and it plays an important role in many physiological and metabolic processes. The active compounds and metabolites of *Hippophae* plants have an antioxidant effect, making them suitable candidates of new functional foods [12]. In addition, *Hippophae* is rich in unsaturated fatty acids; its unsaturated fatty acid content is higher than its saturated fatty acid content, which usually accounts for approximately 58% to 88.9% of the total fatty acids. The content of the seed of *Hippophae* is the highest, which leads to the rich biological activity of sea-buckthorn oil. *Hippophae* plants are also rich in carotenoids, which have anticancer, anti-ulcer and growth-stimulating effects. Therefore, sea buckthorn is a promising plant containing many dietary and medicinal compounds with potential beneficial applications for improving human health [13].

After a thorough literature search, we found that there is no comprehensive literature review on the antioxidant activity and mechanism of polyphenol compounds of *Hippophae* species. In this review, we present a comprehensive overview and the latest information on the chemical constituents, antioxidant activity and application of polyphenols from *Hippophae* species. We also provide a theoretical basis for the further development and utilization of *Hippophae* species.

## 2. Chemical Constituents and Structures of Polyphenols

**Figure 2:**
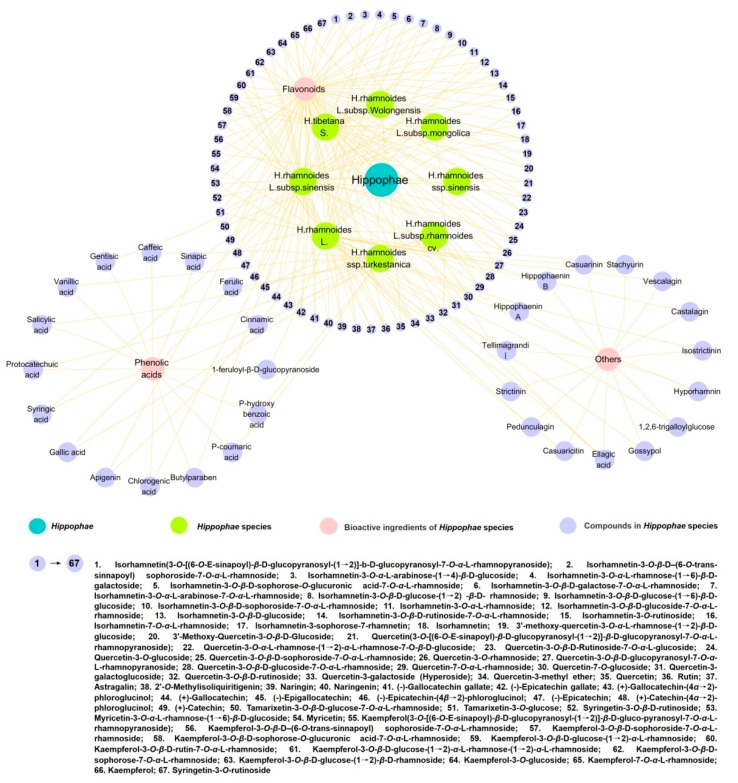
Polyphenolic compounds from the Hippophae species.

Polyphenols in *Hippophae* species are mainly flavonoids and phenolic acids, and research has shown that polyphenol content in the fruit of *H. rhamnoides* L. subsp. *wolongensis* ranges from 29.8 to 38.8 mg GAE/g (GAE, gallic acid equivalents) [14], higher than that in mulberry (4.44 mg GAE/g) [15], pomegranate (3.90 mg GAE/g), red raspberries (3.00 mg GAE/g), blueberry (8.40 mg GAE/g) and blackberry (7.40 mg GAE/g) [16]. In addition, it has been reported that the total polyphenol content of sea buckhorn leaf (*H. rhamnoides* L., Korea) and green tea extracts (young tea leaves harvested in Jeju Island, Korea) ranged from 23.0 to 66.0 mg GAE/g and from 33.0 to 118.0 mg GAE/g, respectively [17]. However, compared with those of green tea from other regions (37.4 mg GAE/g; fresh tea leaves harvested in Guizhou Province, China), the polyphenol content of sea buckthorn also has some advantages [18]. Although the polyphenol contents in berries and leaves vary depending on the species, geographical location and the degree of physiological maturity, they are the most important active ingredients contributing to the antioxidant activities of *Hippophae* species. Nearly 100 polyphenolic compounds have been isolated and identified from the *Hippophae* species, as shown in Figure 2.

### 2.1. Flavonoids

Flavonoids are natural polyphenol compounds widely distributed in *Hippophae* species. They are mostly found in the fruits and leaves of the plant. According to their parent nuclear, flavonoids are divided into flavonoids, flavanols and flavanones in *Hippophae* species. Isorhamnetin, quercetin and kaempferol [19] are the main flavonoids found in *Hippophae* species. 

**Figure 3:**
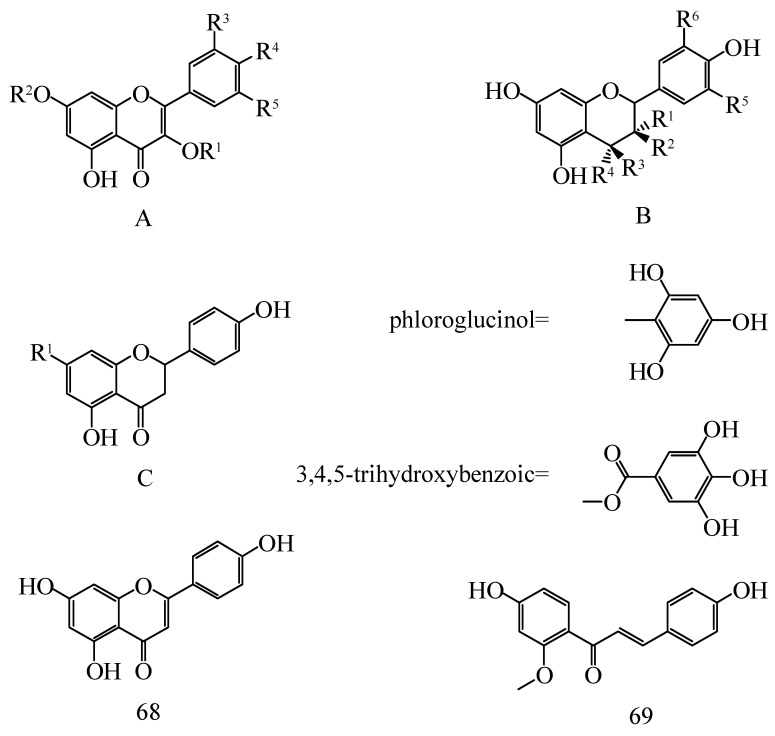
Chemical structures of flavonoids from *Hippophae* species.

The flavonoids (vitamin P) of *Hippophae* species are often used as inhibitors of ascorbic acid oxidation, especially in treating cardiovascular diseases [20]. Xing [21] found that the total flavonoids in the leaves, pulp, pericarp and seeds of *Hippophae* species were 2.24%, 0.95%, 0.51% and 0.31%, respectively, indicating that the leaves have the highest total flavonoid content. Fu [22] used spectrophotometry to measure and compare the total flavonoid content in the leaves of different *Hippophae* species; the results showed that the total flavonoid content in *H*. *rhamnoides* L. subsp. *sinensis* and *H*. *gyantsensis* were the highest (0.7392% and 0.7814%, respectively), followed by that in *H*. *tibetana* S. and *H*. *rhamnoides* L. subsp. *yunnanensis* (0.5879% and 0.4980%, respectively). The chemical constituents and structures of flavonoids in *Hippophae* species are shown in Figure 3 and Table 1.

### 2.2. Phenolic Acids

**Figure 4:**
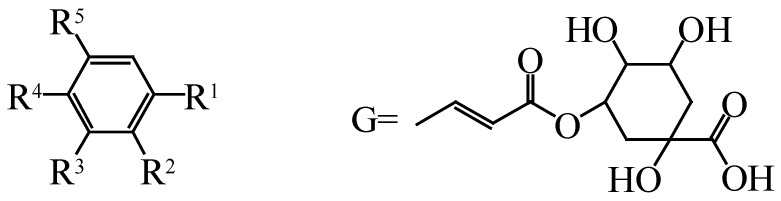
Chemical structures of phenolic acids from *Hippophae* species.

Phenolic acids are the organic acids containing an aromatic ring; these compounds are abundant in *Hippophae* species in the form of free radicals, or bound as esters and glycosides [27]. Phenolics are the major compounds of *Hippophae* plants with antioxidant activity [38]. They are divided into hydroxybenzoic acid, hydroxycinnamic acid, and their derivatives, according to the number and position of the hydroxyl and methoxy groups in their aromatic ring. The total phenolic acid content in the seeds of *Hippophae* species is higher than that in the fruits and seed coat. In a study, the total phenolic acid content in the seeds was released as a soluble ester (57.3% of the total phenolic acid); free phenolic acid and phenolic acid released by the glycosidic bond accounted for 8.4% and 34.3% of the total phenolic acid, respectively [35]. Phenolic acids in *Hippophae* species mainly include gallic acid, syringic acid, protocatechuic acid, salicylic acid, vanillic acid, gentisic acid, caffeic acid, sinapic acid, ferullic acid, cinnamic acid, 1-feruloyl-*β*-d-glucopyranoside and chlorogenic acid [12]. Among them, gallic acid is the predominant phenolic acid in both the fruits and leaves of *Hippophae* species. The chemical constituents and structures of phenolic acids in *Hippophae* species are shown in Figure 4 and Table 2.

### 2.3. Others

**Figure 5:**
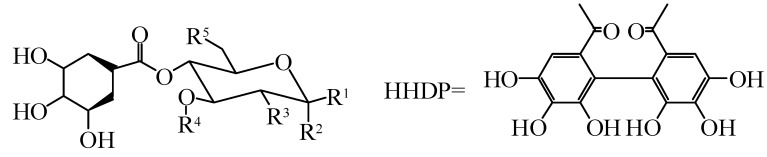
Chemical structures of others from *Hippophae* species.

**Figure 6:**
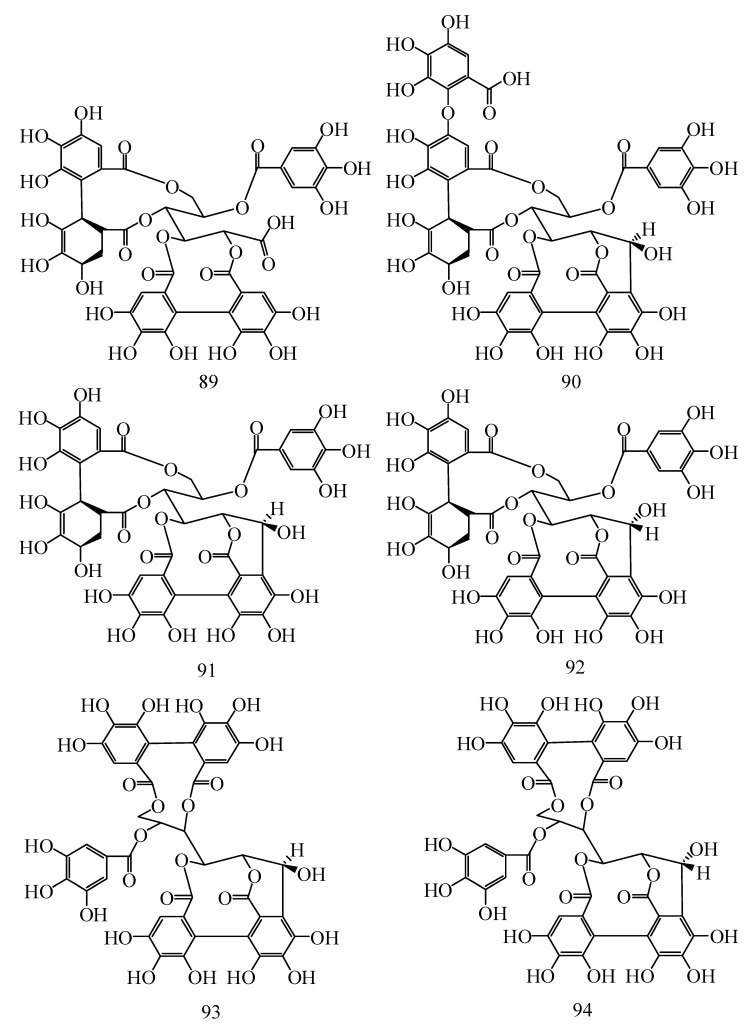

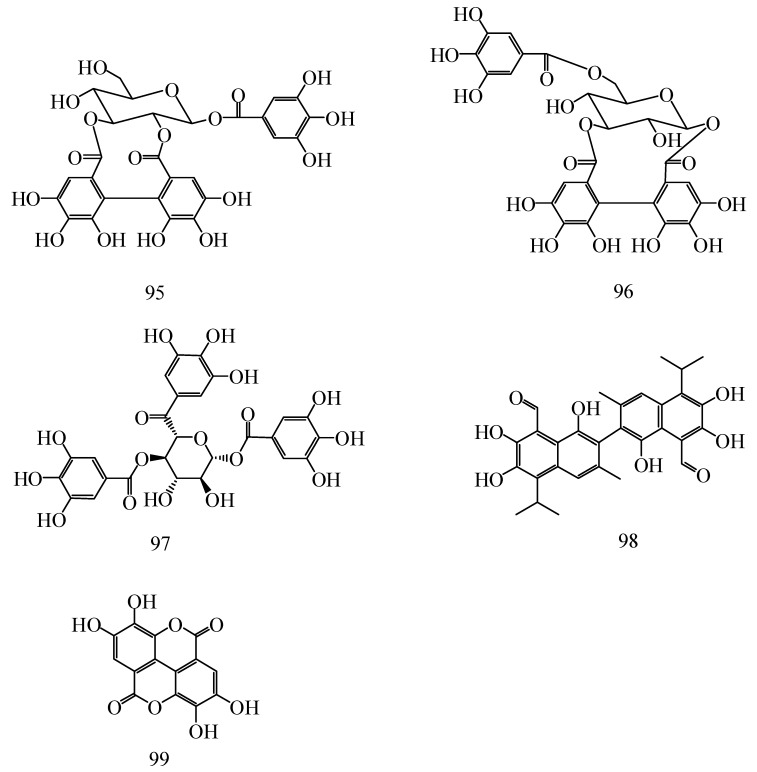
Chemical structures of others from *Hippophae* species.

In addition to flavonoids and phenolic acids, *Hippophae* species contain tannins. Tannins are water-soluble polyphenol compounds found in alkaloids, polysaccharides and proteins with relatively high molecular weight [40]. They can be subdivided into hydrolysable and concentrated tannins. Hydrolyzed tannins include gallic acid esters (gallic acid and ellagic-tannin), and because the molecule has an ester bond and glycosidic bond, it can be hydrolyzed into a small molecule compound and sugar or polyol. Condensed tannins (also known as proanthocyanidins) are polymers of the polyhydroxyflavan-3-alcohol monomer [12]. Their basic structures are formed via the condensation of catechin, (-) epicatechin, and other flavan-3-alcohols or flavan-3,4-glycols at position 4,8- or 4,6 within their carbon–carbon bonds. Tannins isolated from *Hippophae* species mainly include strictinin, 1,2,6-trigalloylglucose, hyporhamnin, isostrictinin, tellimagrandin I, pedunculagin, hippophaenin A, casuaricitin, casuarinin, stachyurin, Hippophaenin B, castalagin and vescalagin. Gossypol, a phenolic aldehyde, was also found in *Hippophae* species. The chemical constituents and structures of tannins in *Hippophae* species are shown in Figure 5 and Figure 6, as well as Table 3 and Table 4.

## 3. Antioxidant Activity

**Figure 7:**
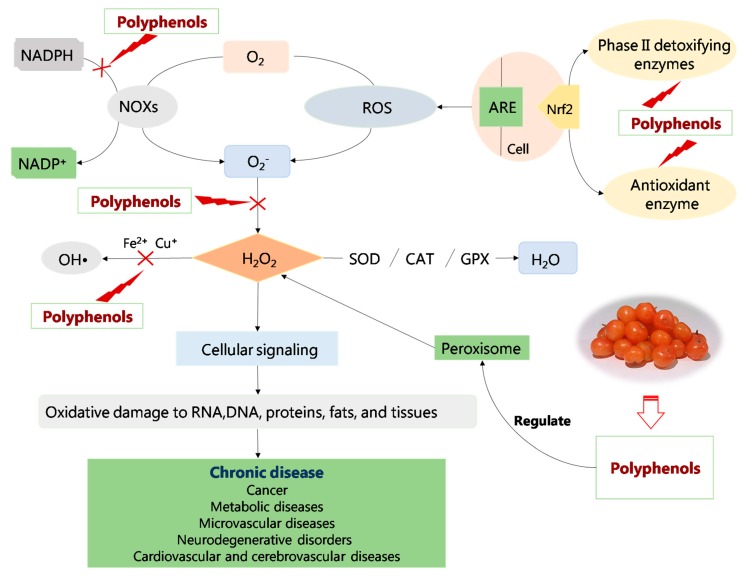
The antioxidant mechanism of polyphenols from *Hippophae* species. (NADPH: nicotinamide adenine dinucleotide phosphate; NADP^+^: nicotinamide adenine dinucleotide phosphate; NOXs: NADPH oxidases; SOD: superoxide dismutase; CAT: catalase; GPX: glutathione peroxidase).

Dietotherapy with antioxidant foods is a very convenient and effective method for the supplementation of endogenous antioxidants to alleviate damage due to free radicals [44]. Phenolic compounds are secondary metabolites with antioxidant activity. Their biological activity depends on their structures, combination with other compounds, solubility, absorption and metabolism. The antioxidant mechanisms of polyphenols from *Hippophae* species (Figure 7) can be summarized as follows.

### 3.1. Regulation of Enzyme Activity

The enzymes related to free radicals are divided into two categories: oxidase and antioxidant enzymes [45]. An antioxidant effect refers to enhancing the activity of antioxidant enzymes and inhibiting the activity of related oxidase. Among antioxidant enzymes, superoxide dismutase (SOD), glutathione peroxidase (GPX) and catalase (CAT) plays an important role in the induction of reactive oxygen-scavenging enzymes, and glutathione (GSH) synthase can induce the synthesis of endogenous antioxidant enzymes. On the contrary, NADPH oxidase (NOX), xanthine oxidase (XO), lipoxygenase (LOX), monoamine oxidase (MAO) and inducible nitric oxide synthase (iNOS) promote the production of ROS. For example, XO can catalyze the oxidation of xanthine and hypoxanthine, thus producing peroxide free radicals [46]. Free radicals can be produced by redox and peroxide of transition metal ions (iron and copper). Therefore, promoting antioxidant enzyme production as well as reducing oxidase and metal ion formation can achieve good antioxidant effect.

#### 3.1.1. Enhancing Antioxidant Enzyme Activity

Endogenous antioxidant enzymes include SOD, GPX, CAT, GSH reductase (GR) and glutathione thiotransferase (GST) [47]. SOD is a very effective antioxidant enzyme that converts decomposed superoxide anions into H_2_O_2_ and O_2_, thereby producing alcohols and water that are harmless to the body. CAT reduces H_2_O_2_ to H_2_O and O_2_, resulting in free radical detoxification. GPX reduces active peroxide to alcohol and water, whereas GSH is oxidized to glutathione disulfide (GSSG). GSSG is reclaimed into GSH by GR. GR is the main enzyme maintaining the glutathione redox state. GST is generally regarded as a 2-phase enzyme, which mainly exists in electrophilic compounds for detoxification [48]. Several studies have also shown that GST can catalyze the decomposition of the lipid hydrogen peroxide produced by oxidative damage of lipid molecules.

Wang et al. treated rabbits with the total flavones from *H. rhamnoides* L. (sea buckthorn) for 2 weeks pre-light exposure and 1-week post-light exposure until sacrifice. The rabbit’s retinal function was assessed by an electroretinogram 1 day before light exposure and on days 1, 3 and 7 post-light exposure. On the seventh day after light exposure, the thickness of the extraretinal nuclear layer and TUNEL were measured to evaluate the degree of retinal degeneration. Western blotting analysis, enzyme-linked immunosorbent assay and immunohistochemistry were used to investigate the antioxidant mechanisms of the total flavones from sea buckthorn in the process of the retinal degeneration induced by visible light. The total flavones from sea buckthorn ameliorated the retinal oxidative stress induced by light exposure, as determined by the measurements of glutathione peroxidase (GSH), CAT, total antioxidant capacity (T-AOC) and malonaldehyde (MDA). Moreover, the activities of GSH and CAT in the retinal injury model group were significantly lower than those in the control group (*p* < 0.05). Overall, the results of this study revealed that total flavones from sea buckthorn have antioxidant effects and indirectly inhibit retinal cell apoptosis [49].

Zhang et al. used *Caenorhabditis elegans* as a model organism to investigate the antiaging effects and mechanism of sea buckthorn seed extract. The samples were prepared using ethanol extraction and macroporous resin purification. The effects of different concentrations of sea buckthorn seed extract on the oxidative stress defense of *C. elegans* were studied. To explore the antiaging mechanism of sea buckthorn seed extract at the gene level, antioxidant enzyme activity and related gene expression were studied. The results showed that sea buckthorn seed extract significantly reduced oxidative stress (*p* < 0.05). In addition, sea buckthorn seed extract increased the activity of SOD, CAT and GSH. The activity of SOD and CAT was significantly higher in the sea buckthorn seed extract-treated group than in the control group. It was concluded that sea buckthorn seed extract improves the activity of antioxidant enzymes, and thus has an antiaging effect [50].

An in-vivo study investigated the antioxidant activities of the phenolic-rich fraction (PRF) of sea buckthorn leaves on oxidative stress induced by CCl_4_ in Sprague–Dawley rats. The total phenol content was 319.33 mg GAE/g PRF, and the amounts of gallic acid, isorhamnetin, quercetin, myricetin and kaempferol were in the range of 1.935–196.89 mg/g, as determined by reverse-phase high-performance liquid chromatography. PRF had a protective effect on liver lipid peroxide, hydrogen peroxide, protein carbonylation, liver GSH decrease and liver antioxidant enzyme (SOD, CAT, GPX, GR, GST) activity induced by CCl_4_. The data obtained in this study suggested that PRF exerts strong antioxidant activity, prevents oxidative damage of main biomolecules, and protects the liver from CCl_4_-induced oxidative damage [48].

#### 3.1.2. Inhibiting the Production of Oxidase

Inducible nitric oxide synthase (iNOS) overexpression leads to the production of a large amount of NO. Under normal conditions, NO has both neuroprotective and neurotoxic effects. Excessively high concentration of endogenous and exogenous NO will lead to the production of OH• and nitrogen dioxide free radicals in large quantities, thus causing damage to proteins, nucleic acids and cell membranes, accelerating mitochondrial damage and promoting cell apoptosis [51,52]. Therefore, inhibition of iNOS expression and the reduction of NO concentration can significantly increase antioxidant capacity and decrease oxidative stress injury. Yang’s research provided evidence that sea buckthorn fruit is an important inhibitor of nitric oxide [53]. 

This study reported the antioxidant effect of sea buckthorn on C6 glioma cells with induced hypoxia. Cytotoxicity as well as nitric oxide and ROS production increased significantly after 12 h of cellular exposure to hypoxia, compared to that in the control group. This resulted in decreased intracellular antioxidant levels and glutathione/oxidized glutathione ratio. The results showed that sea buckthorn pretreatment has a significant inhibitory effect on the production of ROS and nitric oxide induced by hypoxia, showing strong antioxidant activities [54]. It has also been shown that the antioxidant indices of sea buckthorn flavonoids in aging rats induced by D-galactose include monoamine oxidase (MAO) and nitric oxide (NO), in addition to related antioxidant enzymes. The results showed that *H. rhamnoides* L. flavonoids increased the level of related antioxidant enzymes and decreased MAO activity (*p* < 0.05) and NO content (*p* < 0.05) in rats. This study provided evidence of the antioxidant activity of sea buckthorn [55].

Plasma iron and copper are catalysts of many free radical synthetic reactions; especially, iron ions catalyze lipid peroxidation and free radical synthesis. Polyphenols can bond with free metal ions. For example, the catechol structure of the quercetin molecule can bind with Fe^2+^ or Cu^2+^ to form inactive metal complexes, which can reduce oxidation reactions and the damage caused by free radicals to the body [56,57]. In a study, the antioxidant effects of aqueous and hydroalcoholic extracts of sea buckthorn leaves were studied using reversed-phase high-performance liquid chromatography. The total phenol content and total flavonoid content of the leaf extracts was determined by colorimetric analysis. The reducing potential of aqueous and hydroalcoholic sea buckthorn leaf extracts were determined using a modified iron (III) to iron (Ⅱ) reduction assay. The Fe^3+^/ferricyanide complex can be reduced to its ferrous form by the addition of a reducing agent to the solution. Therefore, the addition of Perls’ Prussian blue to the solution creates an insoluble complex with iron (II) that can be measured as a precipitate [58]. The reducing potential of hydroalcoholic sea buckthorn leaf extract is lower than that of ascorbic acid, and higher than that of aqueous sea buckthorn leaf extract. In addition, the reductive activity of each extract was determined to be concentration-dependent. The antioxidant activity of the sea buckthorn extracts was determined using ABTS, DPPH and ferric reducing/antioxidant power (FRAP) assays. The results indicated that both the aqueous and hydroalcoholic sea buckthorn extracts had significant antioxidant activity [59]. Ting et al. induced oxidative stress in mice with carbon tetrachloride (CCl_4_), and then treated them with sea buckthorn seed oil to study the in vitro and in vivo antioxidant capacity of the seed oil. In this study, the ferrous ion chelating ability of the seed oil was determined by reduction of absorbance at 562 nm. The results of a quantitative colorimetric assay revealed that sea buckthorn seed oil at concentrations of 0.92–18.3 mg/mL chelated 7.74%–38.5% of ferrous ions. It was indicated that the sea buckthorn seed oil has a good iron-chelating effect, and has a certain protective effect against oxidative damage [60]. However, it should be pointed out that from the physiological perspective, in-vitro testing of the FRAP reaction in plasma may not reflect the actual physiological structure or activity of the body. Currently, clinical studies are needed to measure and compare changes in FRAP values in relation to specific pathological conditions according to different populations, as well as to monitor FRAP values under different treatment strategies. Despite the limitations, the FRAP experiment appears to be an attractive and potentially useful test. The results are highly reproducible over a wide concentration range. In conclusion, the FRAP experiment can potentially be an effective indicator of antioxidant defense in oxidative stress and related studies [61,62].

### 3.2. Effects on Cell Antioxidant Responses

Nuclear factor erythroid 2-related factor 2 (Nrf2) is a key transcription factor of the cellular antioxidative stress system [63]. Moreover, Nrf2 nuclear translocation combined with antioxidative response element antioxidant responsive element (ARE) on the nucleic acid sequence is a key link in the activation of Nrf2-ARE signaling pathways [64]. Currently, the Nrf2/ARE signaling pathway appears to be the most important endogenous antioxidant stress pathway [65,66]. Furthermore, Nrf2 is pivotal in the host defense mechanism, and numerous key phase II enzymes, such as NQO1 and HO-1 [67], are controlled by Nrf2 transcription factor under ROS conditions [4]. 

Research has been conducted on paraquat (PQ)-exposed A549 cells to evaluate the antioxidant effects of *H. rhamnoides* L. (sea buckthorn) extract to determine whether sea buckthorn extract induces the expression of Nrf2, its downstream target genes, and other antioxidant-related genes, including Nrf2 target genes, such as NQO1 and HO-1, and phase II detoxifying genes, including GPx1, GSR, SOD1, CAT, PRDX1 and LPO [68]. The results showed that pretreatment of A549 cells with sea buckthorn extract (25–200 µg/mL) significantly attenuated PQ (200 µM)-induced cellular toxicity. Moreover, sea buckthorn extract was shown to effectively induce Nrf2 gene expression in a concentration-dependent manner, which significantly contributed to protection against PQ-induced cell death. Notably, Nrf2 expression was markedly induced by sea buckthorn treatment only. In addition, the accumulation of Nrf2 was observed following treatment with sea buckthorn extract. Flavonoid compounds present in the sea buckthorn extract may be involved in the activation of Nrf2, as it has been shown that synthetic flavonoid compounds are potent inducers of the ARE/Nrf2/Keap1 signaling pathway [69]. Based on these results, it was hypothesized that sea buckthorn extract may be used as a potential therapeutic agent for the treatment of various oxidative stress-related diseases.

Studies have shown that Nrf2 is also a major regulator that modulates the expression of the phase II detoxifying enzymes SOD and GPx [70]. Induction of phase II enzymes that neutralize reactive electrophiles and act as indirect antioxidants is an important mechanism of protection against many diseases [71]. The antioxidant effect and action mechanism of *H. rhamnoides* L. (sea buckthorn) leaf tea extracts have been reported in H_2_O_2_-induced murine RAW264.7 macrophages (RAW264.7 cells). The results showed that sea buckthorn leaf tea extracts (40 µg/mL) protected RAW264.7 cells from H_2_O_2_ (5 mM)-induced damage. Cells incubated with sea buckthorn leaf tea extracts showed significantly (*p* < 0.05) elevated SOD and GPx activities, compared with H_2_O_2_-treated controls. The analyses showed that sea buckthorn leaf tea extracts also upregulated the expression of Nrf2; the activation of the Nrf2/ARE antioxidant signaling pathway might play an important role in rescuing cell viability. However, further research is necessary to determine whether elevated Nrf2 expression results in an increase in antioxidant enzyme activity [17].

Furthermore, the antioxidant effect of dietary *H. rhamnoides* L. (sea buckthorn) pomace in ram testis and epididymis was investigated. Rams received diet containing different levels of sea buckthorn pomace (0%, 10%, 20% and 30%) for 65 days. Testis and epididymis samples were collected, and the mRNA and protein expression of antioxidant enzymes was detected. The results showed that supplementation of 20% sea buckthorn pomace reduced the mRNA expression of GPx4 in the testis, Cu–ZnSOD and GPx4 in the caput epididymis, CAT in the corpus epididymis, and Cu–ZnSOD in the cauda epididymis, as well as the protein expression of Nrf2 in the caput epididymis and Cu–ZnSOD in the cauda epididymis [72].

### 3.3. Others

In addition to the mechanisms mentioned above, lipid peroxidation and free radical-scavenging activity have also been identified as antioxidant mechanisms of sea buckthorn phenolic compounds.

Lipid peroxidation is mainly caused by ROS. A free radical chain reaction occurs in the biological membrane phosphatide and initiates propagation reactions, which lead to the damage of erythrocyte membranes and consequently, hemolysis [73,74]. In biological systems, lipid peroxidation produces many aldehydes, among which MDA is the most important indicator of lipid peroxidation. Lipid peroxidation can decrease cell membrane fluidity, inactivate membrane binding proteins, and create cytotoxic aldehydes, such as malondialdehyde or 4-hydroxynonenal. Water-soluble peroxides may react with metals to produce other kinds of free radicals, which can also cause membrane damage [48]. Recent studies have suggested that sea buckthorn has a potent inhibitory effect on lipid peroxidation [75]. This effect might be attributed to its high polyphenolic compound content [76]. In a study, Sprague–Dawley rats were pretreated with the total flavonoid from sea buckthorn by gavage, and then subjected to myocardial ischemia-reperfusion injury induction. The results showed that after 40 min of ischemia and 30 min of reperfusion, the total flavonoids significantly reduced pathological changes in the ultrastructure of the ischemia-reperfusion injury area, significantly improved SOD activity in rat cardiac tissue, and reduced MDA generation. 

All these results indicated that the total flavonoids significantly reduced the lipid peroxidase metabolite MDA and inhibited lipid peroxidation in vivo. This inhibitory effect may be caused by the direct scavenging effect on O_2_, suggesting that the total flavonoids provide protection from myocardial ischemia-reperfusion injury by increasing the free radical-scavenging enzyme and inhibiting lipid peroxidation [77]. Liu [78] observed the effect of sea buckthorn extract on lipofuscin in the brain tissue, and lipid peroxidation (via measurement of the MDA level) in the serum of elderly Wistar rats after administration of the extract for 30 days. The results showed that lipofuscin and MDA content in the experimental group was lower than that in the control group. The reason may be that the sea buckthorn extract contained SOD with high activity, which can metabolize lipid peroxide into hydroxyl compounds to protect cells from peroxidative damage.

Free radicals are extremely active, and are also unstable groups of atoms that constantly seize electrons from other atoms for stability, causing an oxidation reaction. Normal biological oxidation and the resulting free radicals can benefit the body by regulating signal transduction between cells, regulating cell formation, inhibiting the viruses and bacteria that enter the body, and preventing infection. Ideally, the concentration of free radicals in the body are maintained in a state of equilibrium by balancing the formation and elimination of free radicals. However, this balance is difficult to maintain owing to disruption by the body’s own bodily activities, emotional fluctuations, and various external environmental factors [79]. Polyphenol compounds can remove free radicals by acting as an electron donor, thus preventing tissue damage caused by free radicals and peroxides [80]. Su [81] studied the antioxidant capacity of ethanol extracts from sea buckthorn berries; the antioxidant capacity was comprehensively investigated by 2,2-azinobis-(3-ethylbenzothiazole-6-sulfonate, nitrogen radical cation) (ABTS) radical-scavenging activity in vitro. The results showed that the ethanol extract of sea buckthorn had a good scavenging effect on ABTS free radicals. At a concentration of 5 mg/mL, the ABTS-scavenging ability of the extract was similar to that of the positive control butylated-hydroxy-toluene (BHT) at 5 mg/mL. In addition, Varshneya evaluated the antioxidant activity of 100% methanolic extract (ME), 70% aqua-methanolic extract (AME) and 100% aqueous extract of the sea buckthorn by-product. The total phenolic content in AME was significantly higher than that in other extracts. All extracts scavenged different radicals in vitro in a concentration-dependent manner. AME had the lowest IC_50_ values for ABTS, 2,2-diphenyl-1-picrylhydrazyl, superoxide and nitric oxide radicals, whereas ME had the lowest values for hydroxyl radicals. The reducing power of the extracts increased in a concentration-dependent manner, with AME showing the highest reducing power [82].

However, at present, evaluation of free radical-scavenging activity has been focused in vitro, such as in cell models. Moreover, animal studies on this activity and its mechanisms are not in-depth. A commonly used method to evaluate free radical-scavenging activity is the DPPH assay; however, this assay does not provide meaningful information on the actual reactivity of an antioxidant [63]. Some studies have pointed out that free radical-scavenging activity is ineffective in vivo; thus, further studies are necessary [83]. Table 5 shows recent studies on the mechanism of the antioxidant activities of phenolic acids in *Hippophae* species.

## 4. Application

The fruits and leaves of *Hippophae* species are rich in various bioactive components and nutritional ingredients, which are useful in the fields of healthcare, the food industry and the cosmetic industry. In recent years, researchers in the fields of nutrition, food science, medicine, sports science, agriculture and forestry have performed numerous studies on *Hippophae* species, supporting its use as a medicine and food. They believe that the leaves, fruits and seeds of *Hippophae* plants will become ideal high-grade raw materials of nutritious health food with great ecological, social and economic benefits.

### 4.1. Medicinal Values of Hippophae Species

Studies performed worldwide have found that *Hippophae* species contain rich biologically active components that have important medicinal effects on human health; thus, these species are referred to as “vitamin storehouses” and “mystery fruits” [99]. They have been widely used in the field of medicine, and scientific interest in *Hippophae* species as therapeutic agents is rapidly increasing. Guo [100] developed a fermentation method to enhance the active substances with the intestinal autoimmunity function found in *H. rhamnoides* L. leaves, in which beneficial bacteria is firmly wrapped using a drying technique, thereby enabling it to reach the intestine and exert antioxidant effects. Li [101] formulated a hepatoprotective natural product from *H. rhamnoides* L. by inoculating mixed strains of yeasts and lactic acid bacteria to composite culture medium, which was then subjected to a closed culture to obtain a strain solution. The solution was then mixed with *H. rhamnoides* L. and subjected to closed fermentation before elution to obtain the hepatoprotective natural product. The final product contains a large number of active components of detoxification and antioxidant reactions in the liver, and has the following beneficial effects: oxidation resistance, free radical-scavenging, chemical liver injury repair-promoting and liver function-improving effects.

### 4.2. Food Values of Hippophae Species

The fruit of *Hippophae* species is called a third-generation fruit. *Hippophae* plants contain more than 100 types of compounds; this includes vitamin C content of up to 25 mg/g [35]. As of 2018, there have been more than 200 kinds of products derived from *Hippophae* species, including nonalcoholic beverages, wines, jams, ice creams, candies and natural additive pigments [102]. Zou [103] applied for a patent for the *H. rhamnoides* L. fruit peel powder, which is rich in antioxidant nutrients, as a nutritional supplement that can significantly enhance the body’s free radical-scavenging function and improve antioxidant function in immunocompromised people or people with antioxidant hypofunction. Yan [104] provided a health-promoting edible salt product of *H. rhamnoides* L. that has high nutritional value and exerts excellent antioxidant effects; this product also has a unique taste and is additive-free.

### 4.3. Cosmetic Values of Hippophae Species

Cosmetics made from *Hippophae* plant extracts have been produced abroad; these products have natural skin-nourishing and hair-protecting properties. Cosmetic products made of *Hippophae* plants include shampoo, skin cream and bath soak [102]. Smida [105] evaluated the antioxidant activities of sea buckthorn-based mouthwash; the free radical-scavenging activity of sea buckthorn was assessed by DPPH and superoxide anion scavenging (NBT) assays to elucidate the role of sea buckthorn in reducing oxidative stress and damage linked to periodontitis. The results suggested that the superoxide radical-scavenging activities of the sea buckthorn pulp oil-based mouthwash increased markedly with increasing concentrations, suggesting that the mouthwash is a potent scavenger of superoxide radicals. Furthermore, numerous beauty care products derived from *H. rhamnoides* L., such as hair lotions, moisturizing creams, facial creams and facial masks, are popular in Russia [106].

## 5. Conclusions

In recent years, considering the frequent use of *Hippophae* species in traditional Chinese medicine, it is necessary to explore polyphenols more comprehensively. This paper systematically reviewed the composition and antioxidant activity of polyphenols in *Hippophae* species, and provided a basis for the further development and utilization of resources from *Hippophae* species. Although phytochemical and pharmacological studies of *Hippophae* species have made great progresses, there are still many problems in the research and development of *Hippophae* species.

Firstly, there are many subspecies of *Hippophae* plants, which indicates differences in chemical composition among *Hippophae* subspecies; however, there is no systematic study of *Hippophae* subspecies. Secondly, although there are many studies on the chemical constituents of *Hippophae* species, there are few studies on their antioxidant pharmacology at the level of chemical monomers, and the material basis of their pharmacological effect is not clear. Through literature study on the antioxidant activity of *Hippophae*, it was found that the DPPH assay is used in a large proportion of experiments verifying the antioxidant activities of *Hippophae*. However, some studies have shown that the kinetic analysis of DPPH as an antioxidant activity test has been questioned; especially, the significance of antioxidants detected by DPPH in the human body needs to be further confirmed [63]. In addition, the establishment of animal models has greatly contributed to the development of medicines. However, despite the similarities, there are still some differences between humans and animals. Whether the same drug would have the same effect in humans as in animals remains to be elucidated. Therefore, in vitro and animal experiments should be conducted in drug studies to provide a foundation for further development in clinical research [107]. Thirdly, *H. rhamnoides* L. oil and flavonoids are of great demand in the market, but their finished products have poor stability and low purity, limiting their further development and utilization; therefore, other parts of *H. rhamnoides* L. with medicinal values need to be further studied [26].

Our review showed that *Hippophae* species are important plants containing valuable chemicals and important nutrients, and thus can be marketed commercially as alternative sources of nutrition. The commercial and ecological potential of *Hippophae* species can improve people’s living standards and protect the environment. A large amount of experimental data showed that *Hippophae* species have important activity and numerous biologically active substances. Therefore, *Hippophae* species are precious sources of important substances and nutrients with a broad market potential [8].

## Figures and Tables

**Table 1:** Flavonoids from *Hippophae* species.

No.	Compounds	Skeletons	R^1^	R^2^	R^3^	R^4^	R^5^	R^6^	Species	Ref.
1	Astragalin	A	Glu	H	H	OH	H	-	*H*. *rhamnoides* L.	[23]
2	Rutin	A	Rut	H	H	OH	OH	-	*H*. *rhamnoides* L. subsp. *sinensis*, *H*. *tibetana* S., *H*. *rhamnoides* L.	[24,25]
3	Quercetin	A	H	H	H	OH	OH	-	*H*. *rhamnoides* L. subsp. *Wolongensis*	[14]
4	Quercetin-3-methyl ether	A	Me	H	H	OH	H	-	*H*. *rhamnoides* L.	[26]
5	Quercetin-3-galactoside (Hyperoside)	A	Gal	H	H	OH	OH	-	*H*. *rhamnoides* L.	[27]
6	Quercetin-3-*O*-*β*-d-rutinoside	A	Rut	H	H	OH	OH	-	*H*. *rhamnoides* L. subsp. *mongolica*	[28,29]
7	Quercetin-3-galactoglucoside	A	Gal	H	-	H	OH	-	*H*. *rhamnoides* L. subsp. *sinensis*	[28]
8	Quercetin-7-*O*-glucoside	A	H	Glu	H	OH	OH	-	*H*. *rhamnoides* L. subsp. *sinensis*, *H*. *tibetana* S.	[25,26,30]
9	Quercetin-7-*O*-*α*-*l-*rhamnoside	A	H	Rha	H	OH	OH	-	*H*. *rhamnoides* L. subsp. *sinensis*, *H*. *tibetana* S.	[25]
10	Quercetin-3-*O*-*β*-d-glucoside-7-*O*-*α*-L-rhamnoside	A	Glu	Rha	H	OH	OH	-	*H*. *rhamnoides* L. subsp. *sinensis*	[31]
11	Quercetin-3-*O*-*β*-d-glucopyranosyl-7-*O*-*α*-L-rhamnopyranoside	A	Glu	Rha	H	OH	OH	-	*H*. *rhamnoides* L.	[23]
12	Quercetin-3-*O*-rhamnoside	A	Rha	H	H	OH	OH	-	*H*. *rhamnoides* L. subsp. *sinensis*, *H*. *tibetana* S.	[32]
13	Quercetin-3-*O*-*β*-d-sophoroside-7-*O*-*α*-l-rhamnoside	A	Sop	Rha	H	OH	OH	-	*H*. *rhamnoides* L. subsp. *sinensis*, *H*. *tibetana* S.	[25]
14	Quercetin-3-*O*-glucoside	A	Glu	H	H	OH	OH	-	*H*. *rhamnoides* L. subsp. *sinensis*	[29]
15	Quercetin-3-*O*-*β*-d- Rutinoside-7-*O*-*α*-l-glucoside	A	Rut	Glu	H	OH	OH	-	*H*. *tibetana* S.	[25]
16	Quercetin-3-methyl ether	A	OCH3	H	H	OH	H	-	*H*. *rhamoides* L.	[26]
17	Quercetin-3-*O*-*α*-l-rhamnose-(1→2)-*α*-l-rhamnose-7-*O*-*β*-d-glucoside	A	Rha-Rha	Glu	OH	OH	H	-	*H*. *rhamnoides* L. subsp. *sinensis*, *H*. *tibetana* S.	[25]
18	Quercetin(3-*O*-[(6-*O*-E-sinapoyl)-*β*-d-glucopyranosyl-(1→2)]-*β*-d-glucopyranosyl-7-*O*-*α*-l-rhamnopyranoside)	A	Glc-(2′′-*O*-Glc)-(6′′′-*O*-E-sinapoyl)	Glu	OH	OH	H	-	*H*. *rhamnoides* ssp. *sinensis*	[33]
19	3’-Methoxy-Quercetin-3-*O*-*β*-d-Glucoside	A	Glu	H	OMe	OH	H	-	*H*. *tibetana* S.	[34]
20	3’-methoxy-quercetin-3-*O*-*α*-l-rhamnose-(1→2)-*β*-d-glucoside	A	Rha-Glu	H	OMe	OH	H	-	*H*. *tibetana* S.	[34]
21	Isorhamnetin	A	H	H	OMe	OH	H	-	*H*. *rhamnoides* L. subsp. *Wolongensis*, *H*. *rhamnoides* L. subsp. *sinensis*, *H*. *tibetana* S.	[14,25]
22	Isorhamnetin-3-sophorose-7-rhamnetin	A	Sop	Glu	H	H	OMe	-	*H*. *rhamnoides* L. subsp. *sinensis*	[28]
23	Isorhamnetin-7-*O*-*α*-l-rhamnoside	A	H	Rha	H	OH	OMe	-	*H*. *rhamnoides* L.	[26]
24	Isorhamnetin-3-*O*-rutinoside	A	Rut	H	H	OH	OMe	-	*H*. *rhamnoides* L. subsp. *sinensis*, *H*. *tibetana* S.	[25,29,32]
25	Isorhamnetin-3-*O*-*β*-d-rutinoside-7*-O*-*α*-l-rhamnoside	A	Rut	Rha	H	OMe	OH	-	*H*. *rhamnoides* L. subsp. *sinensis*, *H*. *tibetana* S.	[25]
26	Isorhamnetin-3-*O*-*β*-d-glucoside	A	Glu	H	H	OMe	H	-	*H*. *rhamnoides* L. subsp. *sinensis*, *H. tibetana* S.	[25]
27	Isorhamnetin-3-*O*-*β*-d-glucoside-7-*O*-*α*-L-rhamnoside	A	Glu	Rha	H	OH	OMe	-	*H*. *rhamnoides* L. subsp. *sinensis*, *H*. *tibetana* S.	[25]
28	Isorhamnetin-3-*O*-*α*-l-rhamnoside	A	Rha	H	H	OH	OMe	-	*H*. *rhamnoides* L.	[26]
29	Isorhamnetin-3-*O*-*β*-d-sophoroside-7-*O*-*α*-l-rhamnoside	A	Sop	Rha	H	OH	OMe	-	*H*. *rhamnoides* L. subsp. *sinensis*, *H*. *tibetana* S.	[29]
30	Isorhamnetin-3-*O*-*β*-d-glucose-(1→6) -*β*-d-glucoside	A	Glu- Glu	OH	OMe	OH	H	-	*H*. *rhamnoides* L.	[26]
31	Isorhamnetin-3-*O*-*β*-d-glucose-(1→2) -*β*-d- rhamnoside	A	Glu- Glu	Rha	OMe	OH	H	-	*H*. *rhamnoides* L. subsp. *sinensis*	[29]
32	Isorhamnetin-3-*O*-*α*-l-arabinose-7-*O*-*α*-L-rhamnoside	A	Ara	Rha	OMe	OH	H	-	*H*. *rhamnoides* L. subsp. *sinensis*, *H*. *rhamnoides*	[25]
33	Isorhamnetin-3-*O*-*β*-d-galactose-7-*O*-*α*-L-rhamnoside	A	Gal	Rha	OMe	OH	H	-	*H*. *rhamnoides* L. subsp. *sinensis*, *H. rhamnoides* L.	[35]
34	Isorhamnetin-3-O-*β*-d-sophorose-*O*-glucuronic acid-7-*O*-*α*-l-rhamnoside	A	Sop-*O*-GA	Rha	OMe	OH	H	-	*H*. *tibetana* S., *H*. *rhamnoides* L.	[25]
35	Isorhamnetin-3-*O*-*α*-l-rhamnose-(1→6)-*β*-d-galactoside	A	Rha-Gal	H	OMe	OH	H	-	*H*. *rhamnoides* L.	[26]
36	Isorhamnetin-3-*O*-*α*-l-arabinose-(1→4) -*β*-d-glucoside	A	Ara- Glu	H	OMe	OH	H	-	*H*. *rhamnoides* L.	[26]
37	Isorhamnetin-3-*O*-*β*-d–(6-*O*-trans-sinnapoyl) sophoroside-7-*O*-α-l-rhamnoside	A	(6-*O*-trans-sinnapoyl) Sop	Rha	OMe	OH	H	-	*H*. *rhamnoides* L. subsp. *sinensis*	[31]
38	Isorhamnetin(3-*O*-[(6-*O*-E-sinapoyl)-*β*-d-glucopyranosyl-(1→2)]-b-d-glucopyranosyl-7-*O*-a-L-rhamnopyranoside)	A	Glc-(2′′-*O*-Glc)-(6′′′-*O*-E-sinapoyl)	Glu	OMe	OH	H	-	*H*. *rhamnoides* ssp. *sinensis*	[33]
39	syringetin-3-*O*-rutinoside	A	Rut	H	OMe	OH	OMe	-	*H*. *rhamnoides* L. subsp. *sinensis*	[29]
40	Kaempferol	A	H	H	H	OH	H	-	*H*. *rhamnoides* L. subsp. *Wolongensis*	[14]
41	Kaempferol-7-*O*-*α*-l-rhamnoside	A	H	Rha	H	OH	H	-	*H*. *rhamnoides* L. subsp. *sinensis*, *H*. *tibetana* S.	[29]
42	Kaempferol-3-*O*-glucoside	A	Glu	H	H	OH	H	-	*H*. *rhamnoides* L. subsp. *sinensis*, *H*. *tibetana* S.	[25]
43	Kaempferol-3-*O*-*β*-d-glucose-(1→2)-*β*-d-rhamnoside	A	Glu-Glu	Rha	OMe	OH	H	-	*H*. *rhamnoides* L. subsp. *sinensis*,	[29]
44	Kaempferol-3-*O*-*β*-d-sophorose-7-*O*-*α*-l-rhamnoside	A	Sop	Rha	H	OH	H	-	*H*. *rhamnoides* L. subsp. *sinensis*, *H*. *tibetana* S., *H*. *rhamnoides* L.	[25,28]
45	Kaempferol-3-*O*-*β*-d-glucose-(1→2)-*α*-L-rhamnose-(1→2)-*α*-L-rhamnoside	A	Glu-Rha-Rha	H	H	OH	H	-	*H*. *rhamnoides* L.	[26]
46	Kaempferol-3-*O*-*β*-d-rutin-7-*O*-*α*-L-rhamnoside	A	Rut	Rha	H	OH	H	-	*H*. *rhamnoides* L. subsp. *sinensis*, *H*. *tibetana* S., *H*. *rhamnoides* L.	[25,26]
47	Kaempferol-3-*O*-*β*-d-glucose-(1→2)-*α*-L-rhamnoside	A	GluRha	H	H	OH	H	-	*H*. *rhamnoides* L. subsp. *sinensis*, *H*. *tibetana* S., *H*. *rhamnoides* L.	[25,26]
48	Kaempferol-3-*O*-*β*-d-sophorose-*O*-glucuronic acid-7-*O*-α-L-rhamnoside	A	Sop-*O*-GA	Rha	H	OH	H	-	*H*. *tibetana* S., *H. rhamnoides* L.	[25,26]
49	Kaempferol-3-*O*-*β*-d-sophoroside-7-*O*-*α*-L-rhamnoside	A	Sop	Rha	H	OH	H	-	*H*. *rhamnoides* L. subsp. *sinensis*	[29]
50	Kaempferol-3-*O*-*β*-d–(6-*O*-trans-sinnapoyl) sophoroside-7-*O*-*α*-L-rhamnoside	A	(6-*O*-trans-sinnapoyl) Sop	Rha	H	OH	H	-	*H*. *rhamnoides* L. subsp. *sinensis*	[31]
51	Kaempferol(3-*O*-[(6-*O*-E-sinapoyl)-*β*-d-glucopyranosyl-(1→2)]-*β*-d-gluco-pyranosyl-7-*O*-*α*-L-rhamnopyranoside)	A	Glc-(2′′-*O*-Glc)-(6′′′-*O*-E-sinapoyl)	Glu	H	OH	H	-	*H*. *rhamnoides* ssp. *sinensis*	[33]
52	Myricetin	A	H	H	OH	OH	OH	-	*H*. *rhamnoides* L.	[26]
53	Myricetin-3-*O*-*α*-l-rhamnose-(1→6)-*β*-d-glucoside	A	H	H	OH	OH	H	-	*H*. *rhamnoides* L. subsp. *sinensis*, *H*. *rhamnoides* L.	[25,26]
54	Syringetin-3-*O*-*β*-d-rutinoside	A	Rut	H	OMe	OH	OMe	-	*H*. *rhamnoides* L. subsp. *sinensis*,	[29]
55	Tamarixetin-3-*O*-glucose	A	Glu	H	OH	OH	H	-	*H*. *rhamnoides* L. subsp. *mongolica*, *H*. *rhamnoides* L.	[26,28]
56	Tamarixetin-3-*O*-*β*-d-glucose-7-*O*-*α*-l-rhamnoside	A	Glu	Rha	OH	OH	H	-	*H*. *rhamnoides* L. subsp. *mongolica*	[28]
57	(+)-Catechin	B	H	H	H	H	OH	H	*H*. *rhamnoides* L. subsp. *sinensis*	[23,28]
58	(+)-Catechin-(4*α*→2)-phloroglucinol	B	H	OH	H	phloroglucinol	OH	H	*H*. *rhamnoides* L. subsp. *rhamnoides cv*., *H*. *rhamnoides* L. subsp. *sinensis*	[28,36]
59	(-)-Epicatechin	B	OH	H	H	H	OH	H	*H*. *tibetana* S.	[34]
60	(-)-Epicatechin-(4*β*→2)-phloroglucinol	B	OH	H	phloroglucinol	H	OH	H	*H*. *rhamnoides* L. subsp. *sinensis*	[28]
61	(-)-Epigallocatechin	B	H	OH	H	H	OH	OH	*H*. *rhamnoides* L. subsp. *rhamnoides cv*., *H*. *rhamnoides* L. subsp. *sinensis*	[23,36]
62	(+)-Gallocatechin	B	OH	H	H	H	OH	OH	*H*. *rhamnoides* L. subsp. *rhamnoides cv.*, *H*. *rhamnoides* L. subsp. *sinensis*	[23,36]
63	(+)-Gallocatechin-(4*α*→2)-phloroglucinol	B	H	OH	H	phloroglucinol	OH	OH	*H*. *rhamnoides* L. subsp. *rhamnoides cv*., *H*. *rhamnoides* L. subsp. *sinensis*	[28,36]
64	(-)-Epicatechin gallate	B	H	3,4,5-trihydroxybenzoic	H	H	H	OH	*H*. *rhamnoides* L. subsp. *sinensis*	[26]
65	(-)-Gallocatechin gallate	B	H	3,4,5-trihydroxybenzoic	H	H	OH	OH	*H*. *rhamnoides* L. subsp. *sinensis*	[26]
66	Naringenin	C	H	-	-	-	-	-	*H*. *rhamnoides* L. subsp. *sinensis*	[24]
67	Naringin	C	Rha-Glc	-	-	-	-	-	*H*. *rhamnoides* L. subsp. *sinensis*	[24]
68	2′-*O*-Methylisoliquiritigenin	-	-	-	-	-	-	-	*H*. *rhamnoides* L.	[37]
69	Apigenin	-	-	-	-	-	-	-	*H*. *rhamnoides* L.	[23]

**Table 2:** Phenolic acids from *Hippophae* species.

No.	Compounds	R^1^	R^2^	R^3^	R^4^	R^5^	Species	Ref.
70	Gallic acid	COOH	H	OH	OH	OH	*H*. *rhamnoides* ssp. *turkestanica*	[38]
71	Syringic acid	COOH	H	OMe	OH	OMe	*H*. *rhamnoides* L. subsp. *sinensis*	[39]
72	Protocatechuic acid	COOH	H	OH	OH	H	*H*. *rhamnoides* ssp. *turkestanica*	[38]
73	Salicylic acid	COOH	OH	H	H	H	*H*. *rhamnoides* ssp. *turkestanica*	[38]
74	Vanillic acid	COOH	H	OMe	OH	H	*H*. *rhamnoides* ssp. *turkestanica*	[38]
75	Gentisic acid	COOH	OH	H	OH	OH	*H*. *rhamnoides* L. subsp. *sinensis*	[39]
76	Caffeic acid	CHCHCOOH	H	OH	OH	H	*H*. *rhamnoides* ssp. *turkestanica*	[38]
77	Sinapic acid	CHCHCOOH	H	OMe	OH	OMe	*H*. *rhamnoides* L. subsp. *sinensis*	[39]
78	Ferulic acid	CHCHCOOH	H	OMe	OH	H	*H*. *rhamnoides* ssp. *turkestanica*	[38]
79	Cinnamic acid	CHCHCOOH	H	H	H	H	*H*. *rhamnoides* ssp. *turkestanica*	[38]
80	1-feruloyl-*β*-d-glucopyranoside	CHCHCOOH-Glu	H	H	H	OMe	*H*. *rhamnoides* L.	[32]
81	P-hydroxy benzoic acid	COOH	H	H	OH	H	*H*. *rhamnoides* ssp. *turkestanica*	[38]
82	P-coumaric acid	CHCHCOOH	H	H	OH	H	*H*. *rhamnoides* ssp. *turkestanica*	[38]
83	Butylparaben	COO(CH_2_)_3_CH_3_	H	H	H	H	*H*. *tibetana* S.	[34]
84	Chlorogenic acid	G	H	OH	OH	H	*H*. *rhamnoides* L. subsp. *sinensis*	[39]

**Table 3:** Others from *Hippophae* species.

No.	Compounds	R^1^	R^2^	R^3^	R^4^	Species	Ref.
85	Casuaricitin	*O*-(3,4,5-trihydroxybenzoic)	H	HHDP		*H*. *rhamnoides* L.	[41,42]
86	Pedunculagin	H	OH	HHDP		*H*. *rhamnoides* L.	[41,42]
87	Strictinin	*O*-(3,4,5-trihydroxybenzoic)	H	H	H	*H*. *rhamnoides* L.	[41,42]
88	Tellimagrandi Ⅰ	H	OH	3,4,5-trihydroxybenzoic	3,4,5-trihydroxybenzoic	*H*. *rhamnoides* L.	[41,42]

**Table 4:** Others from *Hippophae* species.

No.	Compounds	Species	Ref.
89	Hippophaenin A	*H*. *rhamnoides* L.	[42]
90	Hippophaenin B	*H*. *rhamnoides* L.	[42]
91	Casuarinin	*H*. *rhamnoides* L.	[42,43]
92	Stachyurin	*H*. *rhamnoides* L.	[42,43]
93	Vescalagin	*H*. *rhamnoides* L.	[42,43]
94	Castalagin	*H*. *rhamnoides* L.	[42,43]
95	Isostrictinin	*H*. *rhamnoides* L.	[41,42]
96	Hyporhamnin	*H*. *rhamnoides* L.	[42,43]
97	1,2,6-trigalloylglucose	*H*. *rhamnoides* L.	[41,42]
98	Gossypol	*H*. *rhamnoides* L.	[43]
99	Ellagic acid	*H*. *rhamnoides* L. subsp. *sinensis*, *H*. *tibetana* S., *H*. *rhamnoides* L.	[25,43]

**Table 5:** Antioxidant activities of phenolic acids in *Hippophae* species.

Ingredients	Model	Treatment	Results	Ref.
Apigenin	Sprague–Dawley rats	10, 15 and 20 mg/kg	Apigenin significantly reduced the lipid hydroperoxides and increased the total antioxidant levels.	[84]
Quercetin	Streptozotocin (STZ)-induced diabetes in rats	15 mg/kg/d	Quercetin plays a protective role by reducing lipid peroxidation, NO production and increasing antioxidant enzyme activity.	[85]
Isorhamnetin	Carrageenan-induced paw edema in Sprague–Dawley rats	10 or 30 mg/kg	The induction of Ho-1 by isorhamin can reduce the production of ROS and significantly increase the nuclear level of Nrf2.	[86]
Kaempferol	Sprague–Dawley rats	15 mmol/L	Kaempferol significantly increased SOD activity and GSH/GSSG ratio, while significantly reduced MDA level.	[87]
Myricetin	Human MCF-7 breast cancer cells	0.0, 0.05, 0.1 and 0.2 µmol	Myricetin decreased ferric ions and cellular ROS, respectively.	[88]
Naringenin	Ethanol-induced rats	50 mg/kg	Naringenin elevated the activities of SOD and catalase in the tissues of ethanol-treated rats, inhibit malondialdehyde and to scavenge hydroxyl groups, increased the activities of GR, GPx and GST.	[89]
Naringin	Isoproterenol (ISO)-induced myocardial infarction (MI) in rats	10, 20, 40 mg/kg	Naringin saw a significant decrease in the levels of lipid peroxidative products and improved the antioxidant status by increasing the activities of antioxidant enzymes and nonenzymatic antioxidants.	[90]
Hyperoside	Hydrogen peroxide-induced V79-4 cells	1, 2.5 and 5 µmol	Hyperoside was shown to possess cytoprotective properties against oxidative stress by scavenging intracellular ROS and enhancing the catalase and glutathione peroxidase activities.	[91]
(+)-Catechin	Mature male Wistar rats were given chlorpyrifos	20 mg/kg	Catechin statistically significantly decreased MDA levels, SOD and CAT activities, while increased GPx and GST activities.	[92]
Rutin	Streptozotocin (STZ)-induced diabetic rats	100 mg/kg	Rutin improved the antioxidant status of diabetic rats by decreasing lipid peroxidative products and increasing enzymic and nonenzymic antioxidants.	[93]
Gallic acid	Streptozotocin-induced diabetic male Wistar rats	10 and 20 mg/kg	Gallic acid significant increasing lipid hydroperoxides, SOD, CAT, GPx activities.	[94]
Syringic acid	Nephrotoxicity was induced in male Wistar albino rats by the administration	50 mg/kg	Syringic acid increased GPx, CAT and SOD activities of renal tissue.	[95]
Vanillic acid	Oral squamous cell carcinomas were induced in each hamster’s buccal pouch (left side only)	200 mg/kg	Vanillic acid significantly restored the SOD, CAT, GPx, GSH, vitamin E, vitamin C to near normal range in DMBA-treated hamsters.	[96]
Chlorogenic acid	Intestinal mitochondrial injury by H_2_O_2_	0, 20, 40, 80, and 160 μmol/L	Chlorogenic acid decreased H_2_O_2_-induced ROS production in a dose-dependent manner, and T-AOC, SOD and GSH activities were also increased.	[97]
Ferulic acid	Streptozotocin-induced diabetic rats	10 and 20 mg/kg	Ferulic acid effectively inhibited the lipid peroxidation and elevated the catalase, superoxide dismutase, glutathione and nitric oxide levels.	[98]
